# Nanoparticles of Titanium and Zinc Oxides as Novel Agents in Tumor Treatment: a Review

**DOI:** 10.1186/s11671-017-2007-y

**Published:** 2017-03-27

**Authors:** Janusz Bogdan, Joanna Pławińska-Czarnak, Joanna Zarzyńska

**Affiliations:** Department of Food Hygiene and Public Health Protection, Faculty of Veterinary Medicine, Warsaw University of Life Sciences - SGGW, Nowoursynowska 159, 02-776 Warsaw, Poland

**Keywords:** Nanotechnology, Nanoparticles, Titanium dioxide, Zinc oxide, Cancer, Photodynamic therapy, Sonodynamic therapy

## Abstract

Cancer has become a global problem. On all continents, a great number of people are diagnosed with this disease. In spite of the progress in medical care, cancer still ends fatal for a great number of the ill, either as a result of a late diagnosis or due to inefficiency of therapies. The majority of the tumors are resistant to drugs. Thus, the search for new, more effective therapy methods continues. Recently, nanotechnology has been attributed with big expectations in respect of the cancer fight. That interdisciplinary field of science creates nanomaterials (NMs) and nanoparticles (NPs) that can be applied, e.g., in nanomedicine. NMs and NPs are perceived as very promising in cancer therapy since they can perform as drug carriers, as well as photo- or sonosensitizers (compounds that generate the formation of reactive oxygen species as a result of either electromagnetic radiation excitation with an adequate wavelength or ultrasound activation, respectively). Consequently, two new treatment modalities, the photodynamic therapy (PDT) and the sonodynamic therapy (SDT) have been created. The attachment of ligands or antibodies to NMs or to NPs improve their selective distribution into the targeted organ or cell; hence, the therapy effectiveness can be improved. An important advantage of the targeted tumor treatment is lowering the cyto- and genotoxicity of active substance towards healthy cells. Therefore, both PDT and SDT constitute a valuable alternative to chemo- or radiotherapy. The vital role in cancer eradication is attributed to two inorganic sensitizers in their nanosized scale: titanium dioxide and zinc oxide.

## Review

### Introduction

During the congress of the American Physical Society in Caltech, on 26th December 1959, an American physicist R. Feynman [1] held a presentation. He was the first to claim the matter can be altered at the level of atoms and particles. That statement, the main thesis of the speech entitled “*There*’*s plenty of room at the bottom*,” earned him the attribute of a forerunner of nanotechnology. In the lecture, R. Feynman suggested that in his day, the only limit to the development of nanotechnology resulted from the lack of sufficiently precise tools and techniques. For the first time, the term “nanotechnology” was applied by a Japanese scientist N. Taniguchi [2] and meant the possibility of material engineering at the nanometers level. A book by K. Drexler [3] “*Nanosystems: molecular machinery*, *manufacturing and computation*” contributed to the popularization of the knowledge in this area. At present, nanotechnology is a rapidly developing multidisciplinary field that combines the latest achievements of many disciplines of science, including biology, chemistry, physics, informative technology, and mechanics. It designs nanomaterials (NMs) and nanoparticles (NPs) with at least one dimension ranging in the scale from 1 to 100 nm [4], confers on them the desired properties, and alters them accordingly. For comparison, the diameters of DNA, viruses, and lymphocytes are 2.5, 30–50, and about 7000 nm, respectively [5]. Nanotechnology, besides biotechnology and genetics, has recently become one of the most highlighted fields of science [6]. It gave rise to the creation of nanomedicine [7]. According to Fakruddin et al. [8], thanks to the achievements of nanotechnology, it will soon be possible to increasingly or even fully control the matter structure; hence, to overcome numerous obstacles, the medicine is nowadays struggling against. For example, by using quantum dots, nanotechnology might greatly assist in an early stage cancer diagnosis and therapy [9]. The regeneration and remodeling of damaged neuronal connections is going to be possible, thanks to dendrimers [10]. Carbon nanotubes can deliver drugs to cells in a more targeted manner [11]. Another example might be NM-based video capsule endoscopy providing high quality images of formerly inaccessible spots, e.g., small bowel [12].

The challenge the nanomedicine is now facing is to find the most effective way of the tumor cells eradication [13]. The main area this discipline is trying to explore is designing systems of targeted drug delivery and release into pathologically altered cells, increasing the drug treatment effectiveness, and limiting their side effects [14, 15]. There are several key attributes of NPs in their performance as drug carriers. One of them is their ability to become conjugated with ligands that are specific to cancer cells and that enable the targeted drug delivery [16–18]. NPs can be also conjugated with monoclonal antibodies, a process preventing them from immunological system response [19]. Moreover, thanks to the possibility of modifying the physicochemical properties of NPs, a smooth endocytosis into the cancer cells can occur [20]. NPs have also found their application in nanodiagnostics as biosensors for an early stage tumor markers detection [21] and in gene therapy as carriers delivering into cancer cells foreign nucleic acids that encode proteins killing the tumor cells [22].

The past decade brought about the discovery that many materials used in a variety of industries exhibit formerly undisplayed catalytic properties, after they have been powdered to NPs and subsequently exposed to ultraviolet (UV) radiation or ultrasound (US) wave. Among such materials, there are titanium dioxide (TiO_2_) and zinc oxide (ZnO) [23–25].

### Nanoparticles of Titanium and Zinc Oxides

#### Size, Surface Area/Volume Ratio, and Surface Chemistry

Nanoparticles of titanium dioxide (TiO_2_ NPs) and zinc oxide (ZnO NPs) are single elements with a diameter of 1 < φ ≤ 100 nm [4]. Their size is one of their key properties. The smaller the NP diameter, the bigger becomes the surface area/volume ratio. A high value of surface area/volume ratio allows a greater number of constituting atoms to be located around the surface of the NPs, a phenomenon making the NPs highly reactive and attributing them with new, unique physicochemical properties [26, 27]. Moreover, the smaller the NP diameter, the longer becomes the blood circulation time [28]. Metal oxide nanoparticles whose diameter ranges between 10 < φ ≤ 100 nm are considered perfect in respect of their medical applications. The NPs with φ ≤ 10 nm are able to deeper penetrate tumors and better accumulate in tumor cells, but they are also toxic to healthy cells. Bigger NPs, in the range of 10 < φ ≤ 100 nm, can only hardly be uptaken by healthy cells while they can easily penetrate tumor cells [29]. Nowadays, thanks to the nanoengineering, it is possible not only to precisely control the size of TiO_2_ and ZnO NPs but also to functionalize their surface. The NP functionalization provides for an easier transport within tissues, creates the NP ability to cross plasma membranes and allows them to be delivered to cancer cells in a targeted manner [30]. Thus, NPs of TiO_2_ and ZnO have become a promising tool in medicine [28–30].

#### Photo and Sonocatalytic Properties

The electronic structure of TiO_2_ and ZnO, both belonging to the family of semiconducting metal oxides, determines their catalytic properties [31]. The semiconductors have a valence band (VB), which is filled with electrons, and an electron-free conduction band (CB). The energy difference (ΔE) between those bands is known as band gap, also referred to as energy gap. The band gap is an equivalent of the energy amount necessary to excite an electron from VB to CB. For nanosized titanium dioxide (nano-TiO_2_, anatase polymorph) and nanosized zinc oxide (nano-ZnO), the width of the energy gap is 3.20 and 3.37 eV, respectively [26, 32]. Both amounts approximate the value of the electromagnetic radiation photon energy with a wavelength of λ < 400 nm. In biological trials, semiconducting metal oxides (e.g., TiO_2_, ZnO) are excited by the UV radiation (photoexcitation), mainly in the near ultraviolet range (UV-A, 315 ≤ λ < 400 nm) [33–38]. Less common is the photoexcitation with a wavelength in the medium ultraviolet range (UV-B, 280 ≤ λ < 315 nm) [33, 34, 36]. The use of far ultraviolet range (UV-C, 100 < λ < 280 nm) as an agent responsible for advanced oxidation processes may be hazardous to human health [37]. Another way to excite semiconductor NPs in aqueous solutions is the application of the US wave (sonoexcitation). The US wave (20 kHz < f ≤ 1 GHz), while propagating in the liquid, causes a twofold effect: a temporary punctual dilution (result of the loss of pressure), facilitating the formation of cavitation bubbles; as well as liquid’s thickening (result of the rise of pressure) that facilitates the bubbles’ collapse. This phenomenon is referred to as acoustic cavitation. It is accompanied by a number of secondary effects, among them sonoluminescence, an emission of sonoluminescent light (mainly UV light) during the implosion of the cavitation bubbles suspended in a liquid [39, 40].

**Fig. 1:**
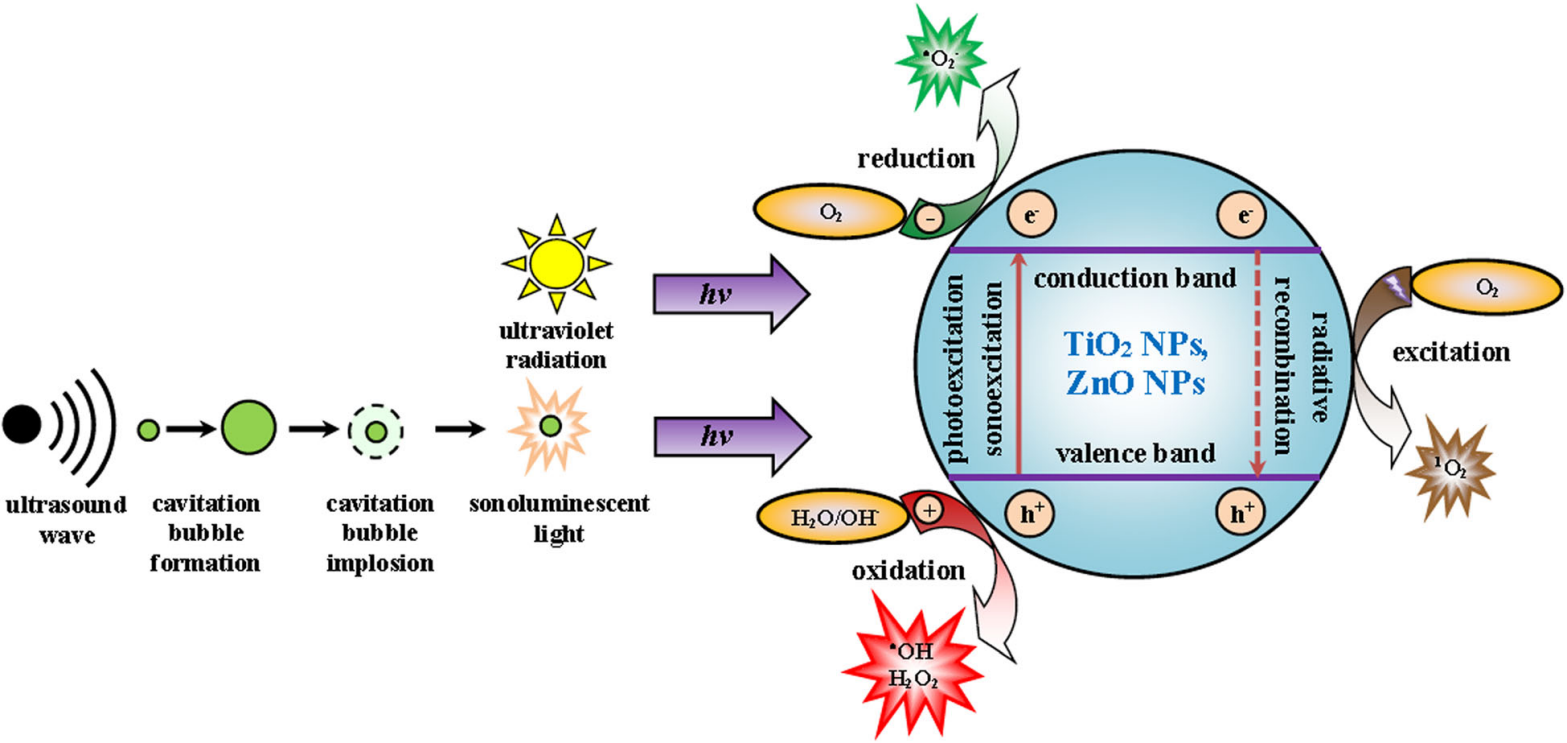
Mechanisms of generating reactive oxygen species (ROS). ROS are generated on the surface of titanium dioxide or zinc oxide nanoparticles (TiO_2_ NPs, ZnO NPs), exposed to ultraviolet (UV) radiation or ultrasound (US) wave in aqueous solutions. Semiconductor NPs can be excited by UV (photoexcitation) or US (sonoexcitation). The acoustic wave, while propagating in the liquid, induces the formation and the subsequent collapse of cavitation bubbles. During their implosion, the emission of the sonoluminescent light takes place. As a result of the TiO_2_ NPs or ZnO NPs photo- or sonoexcitation, electron (e^−^) transfers from valence band to conduction band, leaving behind hole (h^+^). The follow-up process can be twofold. The electrons (e^−^) together with holes (h^+^) react with adjacent molecules (O_2_, H_2_O, OH^−^), generating the formation of ROS (^•^O_2_^−^, ^•^OH, H_2_O_2_). Alternatively, radiative recombination of electron-hole pair (e^−^ + h^+^) results in the emission of photon that transforms the oxygen molecule in the ground state (O_2_) to the oxygen molecule’s excited state, singlet oxygen (^1^O_2_)

The consequence of the semiconductor photo- or sonoexcitation is that electron (e^−^) transfers from VB to CB, leaving behind a positive hole (h^+^). Therefore, a specific “electron-hole” pair (e^−^ + h^+^) is created [40–42]. This bound state, called exciton, lacks stability and exhibits redox properties. Charge carriers (e^−^, h^+^) can migrate to the surface of the catalyst (e.g., TiO_2_ or ZnO) NPs where they can be trapped by adsorbate molecules, initiating thereby the formation of reactive oxygen species (ROS). Excited negative electrons (e^−^) reduce molecular oxygen (O_2_), forming superoxide radical anion (^•^O_2_^−^). Generated positive holes (h^+^) oxidize water molecules (H_2_O) and hydroxide ions (OH^−^), producing hydroxyl radicals (^•^OH) and hydrogen peroxide molecules (H_2_O_2_) [40–43] (Fig. 1).

The inverse process to the generation of charge carriers in semiconductor (e.g., TiO_2_ or ZnO) NPs is the decay of exciton. The recombination of the electron-hole pair (e^−^ + h^+^) is accompanied by the emission of a photon (radiative recombination). In this process, the excitation of ground state oxygen (O_2_) by photon occurs and singlet oxygen (^1^O_2_) is formed [40, 43] (Fig. 1).

### Oxidative Stress and its Anticarcinogenic Effects

The oxidative stress is an underlying factor for adverse biological effects caused by NPs of TiO_2_ and ZnO. In the cells, it is reflected by the increase of the level of ROS and oxidative products, as well as by the depletion of cellular antioxidants [44]. ROS forming is an inherent element of the aerobic cellular metabolism. In their physiological concentrations, ROS perform a series of important functions. They mediate in the intracellular signal transduction [45], regulate protein phosphorylation [46], and control intracellular Ca^2+^ homeostasis [47]. Balance disruptions between the ROS formation and the efficiency of antioxidative systems lead to oxidative stress that poses a serious danger to the integrity of cells and their correct functioning [48].

Eukaryotic cells, similarly to prokaryotic cells, are equipped with mechanisms protecting them from ROS. The first defense line, preventing ROS from forming and from reactions with cell compounds, are proteins (e.g., lactoferrin) that sequestrate metal ions (e.g., Cu, Fe). Interruption of free radical chain reactions is the task of the second defense line that consists of a system of three enzymes: superoxide dismutase (SOD; EC 1.15.1.1), catalase (CAT; EC 1.11.1.6), and glutathione peroxidase (GPX; EC 1.11.1.9), supported by low molecular weight antioxidants, such as glutathione (GSH), thioredoxin (Trx), or coenzyme Q_10_ (CoQ_10_). The third defense line present repair enzymes responsible for nucleic acids damage overhaul, e.g., DNA ligase (EC 6.5.1.1) [48].

ROS can inflict cell injury as an effect of reaction with lipids in cellular membranes [49], nucleotides in DNA [50], or sulfhydryl groups in proteins [51]. Depending on the intensity of the oxidative stress, the cell can undergo carcinogenesis or enter one of the cell death pathways [52]. Antunes and Cadenas [53] proved that as the concentration of certain ROS (e.g., H_2_O_2_) increases, the cell’s viability decreases. In physiological conditions, the concentration of H_2_O_2_ is low and ranges within 5–50 nM. The gradual increase of its quantity in a cell up to the level of 0.7 μM triggers mechanisms that result in a cell proliferation and tumor formation. In case the H_2_O_2_ concentration reaches the value of 1–3 μM, the apoptosis or autophagy (programmed cell death pathways) induction occurs. The concentration in excess of the level of 3 μM becomes toxic for a cell hence leads to its necrosis (unprogrammed cell death pathway). Therefore, the phenomenon of the oxidative stress in tumor cells has recently been used to design new tumor therapies [54, 55].

Increasingly frequent are the opinions that in near future, the NPs of semiconductor metal oxides (e.g., TiO_2_ and ZnO) are going to play the crucial role in medicine as photo- or sonosensitizers applied in ROS-based cancer therapy [56, 57], a finding that naturally corroborates the necessity to study their cyto- and genotoxicity.

### Toxicity of Titanium Dioxide and Zinc Oxide Nanoparticles

Despite the assumption that the microsized titanium dioxide (micro-TiO_2_) is harmless to people and animals [58, 59], the issue of TiO_2_ toxicity has been a research subject in many scientific centers. Recent years have witnessed a dynamic development of nanotechnology. Along with it, there has been an increased concern, though that this compound might be toxic in its NPs form. There are studies proving that NPs (1 < φ ≤ 100 nm) of any compound are more harmful to people and animals than microparticles (MPs) (0.1 < φ ≤ 100 μm) of the same compound [60, 61].

#### Studies In Vivo

Wang and Fan [62] conducted studies showing that the level of the rats’ pulmonary cell impairment, inflicted by the intratracheally instilled TiO_2_, clearly depended upon the diameter of its particles. Kobayashi et al. [63] reported the pulmonary toxicity of TiO_2_ NPs in rats, evaluated on the basis of the short-term post-installation lung injury (up to 7 days after the installation), increased proportionally to the fragmentation of the TiO_2_ particles. Sycheva et al. [64] pointed out the genotoxicity of TiO_2_ NPs (φ = 30 nm), as measured by the number of DNA double-strand breaks and the number of micronuclei, was significantly higher in brain, liver, or bone marrow cells in mice, compared to the genotoxicity of TiO_2_ MPs (φ = 160 nm). Danio rerio, when exposed to TiO_2_ (50 mg dm^−3^) in their NPs form, exhibits more serious oxidative injuries of liver and gill cells, in comparison to the exposition to the MPs form of those compounds [65]. The studies by Ma-Hock et al. [66] showed the rats’ exposition to the aerosol of TiO_2_ NPs (50 mg m^−3^) caused lung inflammation, accompanied by the increase of total neutrophil count in bronchoalveolar lavage (BAL) fluid. Bermudez et al. [67] examined pulmonary responses in rats, mice, and hamsters after their exposition to the aerosol of TiO_2_ NPs (10 mg m^−3^). They concluded there was a clear increase of cytotoxicity markers, i.e., an increase in the total macrophage and neutrophil count in BAL fluid in rats and mice, while in hamsters, there were no distinct changes in the cellular response. Ze et al. [68] proved the TiO_2_ NPs, administered to mice through the nose for 90 days, moved up and built up in the brain, leading to the oxidative stress, excessive glial cells proliferation, and tissue necrosis, as well as inflicting hippocampal cells apoptosis. Lindberg et al. [69] reported that the intraperitoneal administration of TiO_2_ NPs to mice resulted in an increase of DNA damages in leukocytes and in higher micronuclei count in peripheral blood polychromatic erythrocytes. The intratracheal instillation of TiO_2_ NPs showed the same effects in BAL cells. The cyto- and genotoxicity of TiO_2_ NPs were confirmed by many other authors [70–73]. Ferin and Oberdörster [74] showed the dust of TiO_2_ NPs, introduced into the trachea of a rat in the amount of 5 mg per animal, caused respiratory tract cancer within 4 months. Therefore, TiO_2_ NPs in the dust form, contrary to the TiO_2_ MPs in the same form, were classified in the 2B group by the International Agency for Research on Cancer (IARC) [75]. The 2B group assembles compounds that might be carcinogenic for humans.

#### Studies In Vitro

One of the main differences between TiO_2_ MPs and TiO_2_ NPs is a much bigger active surface area of TiO_2_ NPs, a feature resulting in a higher absorption rate of UV radiation and a greater photocatalytic activity [41]. Gurr et al. [76] prove there is a strong correlation between the TiO_2_ NPs genotoxicity and their diameter. TiO_2_ NPs (φ < 50 nm) caused the formation of 8-hydroxy-2-deoxyguanosine (8-OHdG) adducts and the increase of the micronuclei number in human bronchial epithelial cells, while TiO_2_ MPs (φ > 200 nm) in the same concentration were practically harmless. Kongseng et al. [77] report TiO_2_ NPs (φ < 30 nm) were responsible for the rise of the pro-inflammatory cytokine (IL-1β and TNFα) level in human peripheral blood mononuclear cells (PBMCs) in serum-free medium. The in vitro studies of Long et al. [78, 79], conducted on mouse brain microglia cells (BV2 line), proved that TiO_2_ particles with 10 ≤ φ ≤ 20 nm stimulated the generation of ROS and, as a consequence, induced damages of neurons, whereas UV radiation was not a precondition for generating ROS. There are, however, numerous contrary opinions. Fujita et al. [80], drawing on DNA microarray analysis of human keratinocytes (HaCaT line), proved that the natural (with no UV irradiation) toxicity of TiO_2_ NPs was very low. Similar conclusions were presented by Verves and Jha [81] who examined in vitro the toxicity of TiO_2_ NPs (50 mg mL^−1^) to rainbow trout (*Oncorhynchus mykiss*) gonadal tissue cells (RTG-2 line). They reported cyto- and genotoxic effects of TiO_2_ NPs in irradiated cells, only. Saquib et al. [82], while examining the toxicity of TiO_2_ NPs (10 μg mL^−1^) to UV-A irradiated human amniotic epithelial cells (WISH line), noted a decrease in the CAT activity, lowering the GSH level, as well as the arrest of the cellular cycle in the G_2_/M phase. In similar studies on human epidermoid carcinoma cells (A431 line), Shukla et al. [83] observed an increase in lipid hydroperoxide (LPO) concentration, numerous oxidative DNA damages, and the formation of many micronuclei. Vamanu et al. [84] exposed human leukemic monocyte lymphoma cells (U937 line) in vitro to 0.1% colloidal nano-TiO_2_ for 120 min and, subsequently, to UV-A irradiation. After 30 min of UV-A irradiation, significant plasma membrane damages and DNA fragmentation were noticed. Other studies show that TiO_2_ powdered to NPs exhibited ROS-dependent cyto- and genotoxicity towards human HaCaT cells [85], protozoan (*Cryptosporidium parvum*) oocysts [86], as well as towards cysts of *Giardia lamblia* [87] and *Acanthamoeba castellanii* [88], only if the parasites were exposed to UV radiation. The cytotoxicity was determined on the basis of the lysosomal membrane stability, using neutral red retention time (NRRT) assay whereas the genotoxicity was measured by the number of DNA double-strand breaks, using the single-cell gel electrophoresis (SCGE).

ZnO toxicity is also dependent on the scale of its fragmentation [65]. Microsized zinc oxide (micro-ZnO), similarly to micro-TiO_2_, is practically harmless to people and animals [75], contrary to nano-ZnO. Wang et al. [89] studied the toxicity of ZnO NPs to human HaCaT cells and discovered the viability of those cells was clearly reduced, the integrity of their membranes was disrupted, and numerous oxidative DNA damages occurred. Those effects significantly increased when the keratinocytes were exposed to the UV radiation. Similar cyto- and genotoxicity effects of ZnO NPs were observed in human monocytes (THP-1 line) [90, 91] and human lung adenocarcinoma epithelial cells (A549 line) [92], as well as in fish embryos, e.g., *Danio rerio* [93].

Cyto- and genotoxicity of TiO_2_ NPs, as well as of ZnO NPs have been proved in vitro also with many other cells, both healthy, e.g., human dermal fibroblasts [94, 95], human periodontal ligament fibroblasts [96], human alveolar macrophages [97], human gastric epithelial cells [98]; and malignant, e.g., human colonic mucinous adenocarcinoma cells (Ls-174-t line) [99], human colon adenocarcinoma cells (LoVo line) [100, 101], human cervical carcinoma cells (HeLa line) [102–105], human breast adenocarcinoma cells (MDA-MB-468 line) [106], human hepatocellular carcinoma cells (Bel 7402 line) [107], human brain glioblastoma cells (T98G line) [108, 109], or human bladder carcinoma cells (T-24 line) [110].

### Future of Cancer Therapy in View of the Application of Titanium Dioxide and Zinc Oxide Nanoparticles

In recent years, there has been an increase in cancer disease prevalence, an issue inducing an acute need to invent new, effective, and safe therapy methods. In the past decade, nanotechnology and nanomedicine gained quite an importance in anticancer therapy. Those two fields of science joined forces not only to seek substances that would effectively eradicate cancer cells but also to find ways of delivering chemotherapeutics to tumor cells, in a targeted manner [111, 112]. The introduction of nanosized photo- and sonosensitizers to the medicine gave rise to develop new therapeutic strategies, photodynamic therapy (PDT) [113] and sonodynamic therapy (SDT) [114], respectively. Designing of new ways of NM-based targeted drug delivery into cancer cells (targeted therapy) lowered side effects inflicted by many marketed drugs, such as doxorubicin and daunorubicin [115–117]. Among the scientists, great hopes to fight cancer more effectively are attributed to the application of NPs of metal oxides (e.g., TiO_2_, ZnO) in PDT [113, 118, 119], a treatment strategy presenting an alternative to chemo- and radiotherapy.

#### Photodynamic Therapy

**Fig. 2:**
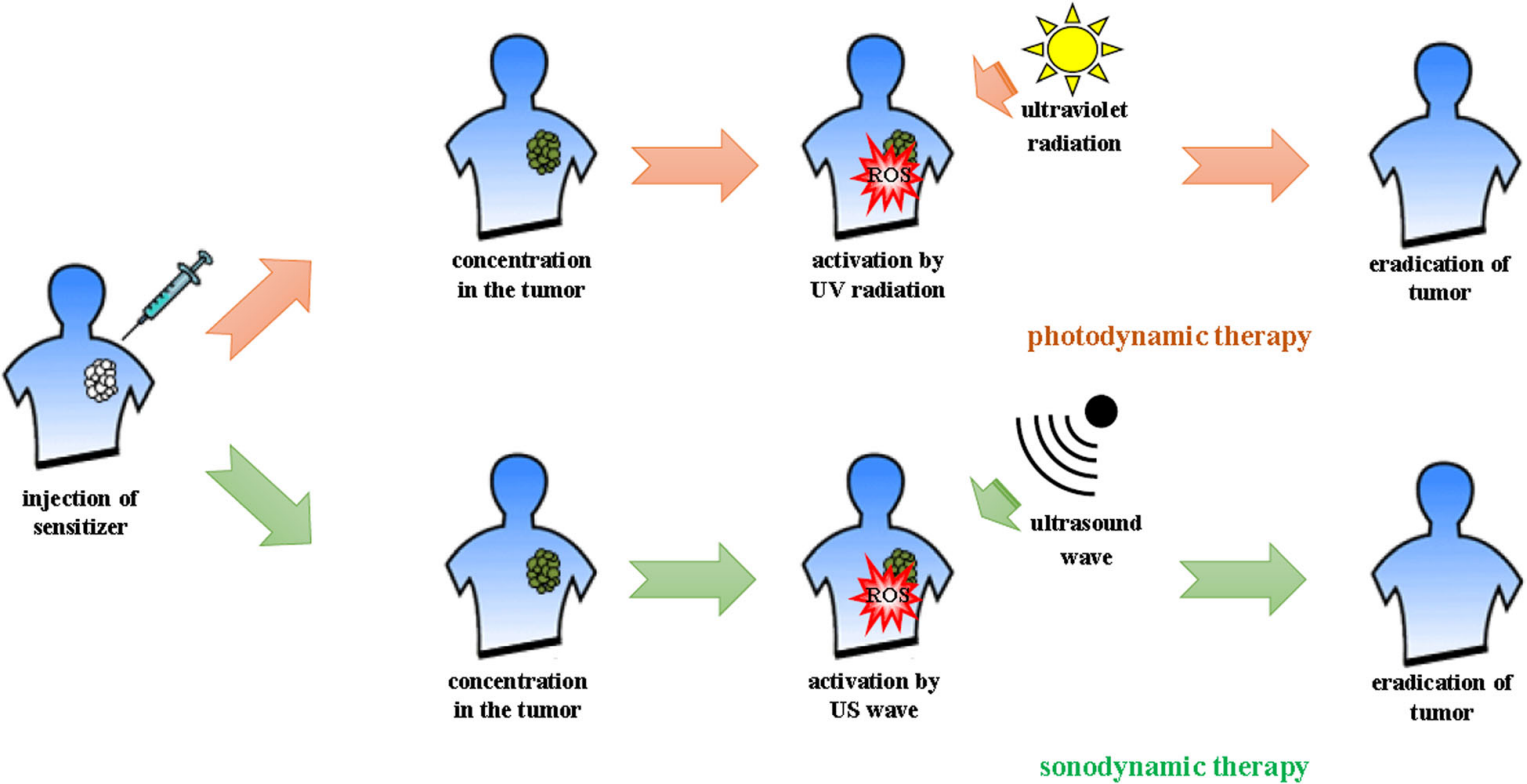
Milestones of photodynamic therapy (PDT) and sonodynamic therapy (SDT) in tumor treatment. Cancer therapies, such as PDT or SDT, build on the administration of sensitive agents into tumor, followed by their activation by UV radiation or US wave, respectively. Activated photo- or sonosensitizers generate ROS that lead to cancer cell eradication

PDT is a treatment in which the administration of photosensitizer to the tumor tissue is followed by the excitation of a photosensitizer by electromagnetic radiation with a specific wavelength, e.g., UV-A or UV-B. Consequently, on the surface of NPs of photoexcited metal oxides, ROS are formed which then damage cancer cells [120] (Fig. 2).

Photosensitizers, such as TiO_2_ or ZnO NPs, can be delivered to the tumor cells as the result of passive or active processes [121]. The passive process takes advantage of the enhanced permeability and retention (EPR) effect. Leaky vasculature in the cancer tissue allows the NPs to be freely uptaken into the tumor cells, a phenomenon leading to the cell death. The uptake takes place also in the inflamed tissue; hence, the passive process does not provide for the targeted NPs delivery to the tumor cells [122]. In the active process, the functionalization of NPs occurs; therefore, the NPs can be delivered to tumor cells in a targeted manner. The functionalization of NPs means their conjugation with specific antibodies or ligands. PDT clearly lowers the cancer therapy side effects, compared to chemo- or radiotherapy [123]. The way the cellular uptake of nanoparticles takes place, their subcellular position and the ability to cause toxic effects depend mainly upon the NPs diameter, their surface area, surface chemistry, and charge [124]. There are two major ways of the NPs uptake into the cell: active uptake by endocytosis and passive uptake by diffusion. Due to their strong hydrophilic properties, both TiO_2_ and ZnO NPs are uptaken into the cells by phagocytosis [125].

**Fig. 3:**
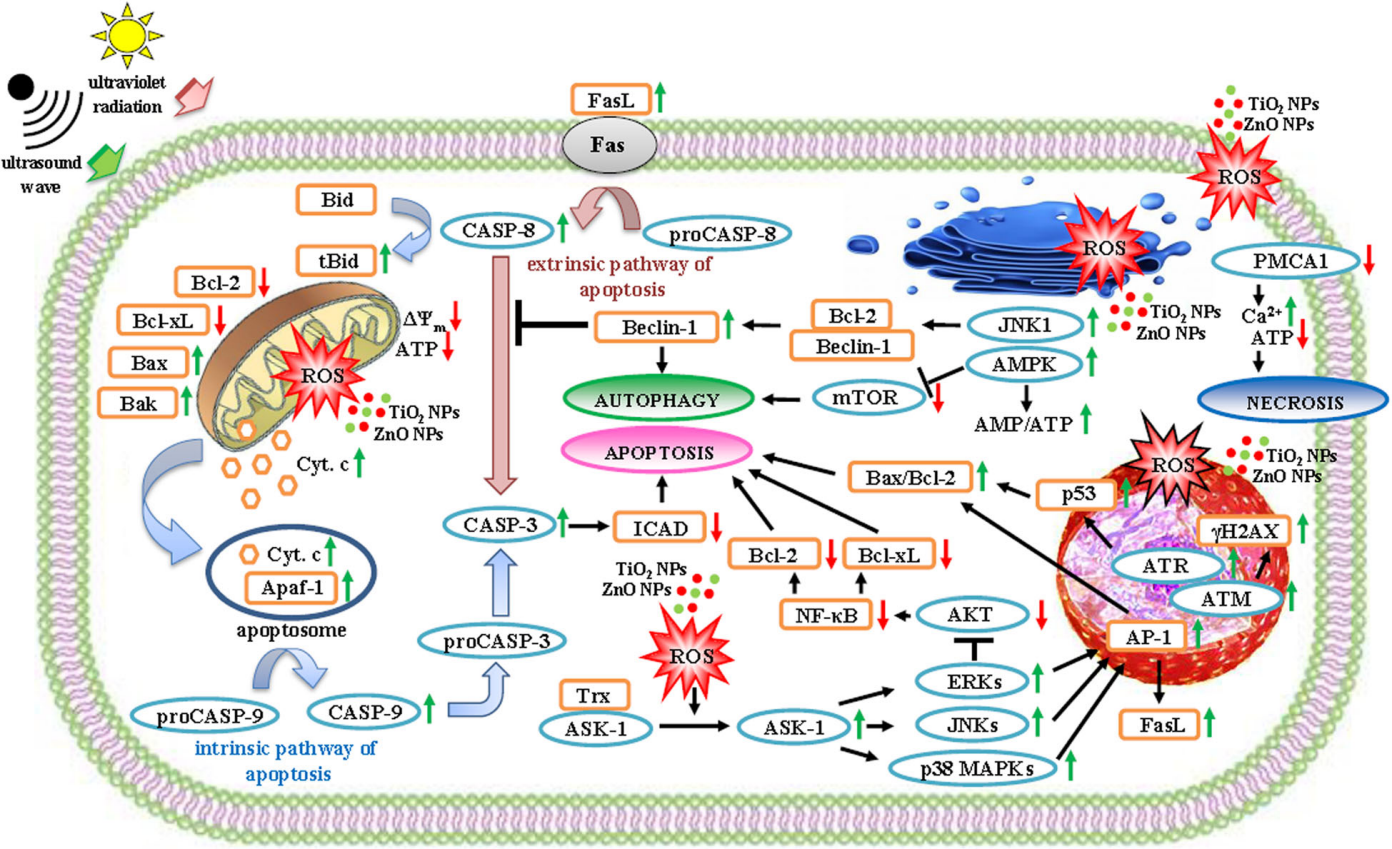
Molecular mechanisms of reactive oxygen species (ROS)-mediated cell death pathways. Activated by ultraviolet (UV) radiation or ultrasound (US) wave, nanoparticles of titanium dioxide (TiO_2_ NPs) and zinc oxide (ZnO NPs) induce ROS-mediated cell apoptosis, autophagy or necrosis. The increase of ROS level in cytoplasm results in thioredoxin (Trx) oxidation and apoptosis signal-regulating kinase 1 (ASK1) activation. ASK1 phosphorylates mitogen-activated protein kinases (ERKs, JNKs, p38 MAPKs) that control the AP-1-mediated synthesis of pro- (Bak, Bax) and anti-apoptotic (Bcl-2, Bcl-xL) proteins, as well as death ligands (FasL), thus promoting the cell apoptosis. Likewise, ERKs promote the cell apoptosis trough attenuation of activity of AKT kinase that controls the NF-κB-mediated synthesis of Bcl-2 and Bcl-xL proteins. The oxidative damages of mitochondrion result in dissipation of mitochondrial membrane potential (ΔΨ_m_), decrease of ATP level and release of cytochrome c (Cyt. c) to the cytosol. Cyt. c, binding with apoptotic protease activating factor 1 (Apaf-1), induces the caspase-dependent apoptosis. The increase of ROS level in nucleus results in the activation of ataxia telangiectasia mutated (ATM), as well as ataxia telangiectasia and Rad 3-related (ATR) kinases that induce the p53-mediated apoptosis and account for the increase of DNA double-strand breaks marker (γH2AX) level. The ROS-induced endoplasmic reticulum stress results in the JNK1-mediated increase of pro-autophagic protein (Beclin-1) level and the AMPK-mediated inhibition of autophagic inhibitor, mammalian target of rapamycin (mTOR) kinase. Both processes lead to the cell autophagy. The oxidative injuries of plasma membrane result in the downregulation of plasma membrane calcium ATPase 1 (PMCA1), in the decrease of ATP level and the increase of Ca^2+^ level. As a consequence, the cell necrosis follows

It is assumed that TiO_2_ NPs, if exposed to the UV radiation, induce, depending on their subcellular position, one of the cell death pathways: apoptosis [105, 126], autophagy [127, 128], or necrosis [129, 130], whereas the mechanism of those processes is still insufficiently examined (Fig. 3).

Zhang et al. [131], having examined UV-excited TiO_2_ NPs toxicity towards human hepatocarcinoma cells (SMMC-7721 line), reported an increase and a decrease of protein levels that are, correspondingly, pro- (e.g., Bax, Bak, tBid) and anti-apoptotic (e.g., Bcl-2, Bcl-xL) molecules. Zhao et al. [132] point out there is an overexpression of the tBid in the TiO_2_ NPs-treated mouse epidermal cells (JB6 line), this protein playing a crucial role in the involvement of mitochondria in oxidative stress-dependent cell death. PDT-induced mitochondria damages result in a selective Bcl-2 degradation, with no effect on the Bax. Therefore, the Bax/Bcl-2 ratio is increased, and the intrinsic pathway of apoptosis is promoted [133–135]. Those effects were noticed in PDT-treated human breast epithelial cells (MCF10A line) [136], human brain glioblastoma cells (U87MG line) [137] or human gastric adenocarcinoma cells (MGC803 line) [138]. Nearly identical changes in levels of proteins regulating apoptosis were observed by Ahamed et al. [139] and by Wahab et al. [140] whereby the purpose of their experiments was to examine the ZnO NPs toxicity towards human A549 cells, as well as human breast adenocarcinoma cells (MCF-7 line) and human hepatocellular liver carcinoma cells (HepG2 line). The level of Bax and Bcl-2 proteins in ZnO NPs-treated (50 μg mL^−1^, 24 h) human HepG2 cells was twofold higher and threefold lower, respectively, compared to control cells [140]. The principle function of Bax is the formation, with the assistance of Bak, of pores in the outer mitochondrial membrane that enhance its permeability. Effectively, the release of cytochrome c (Cyt. c) from the mitochondrial intermembrane space into the cytosol follows. Cyt. c, along with the apoptotic protease-activating factor 1 (Apaf-1), contributes to the formation of the structure called apoptosome. This structure activates the initiator caspase-9 (CASP-9; EC 3.4.22.62) that subsequently activates the executioner caspase-3 (CASP-3; EC 3.4.22.56). Bcl-2 is an inhibitor of apoptosis. It associates with the outer mitochondrial membrane, keeping therefore its integrity [141, 142] (Fig. 3).

According to many authors [139, 140, 143, 144], the key role in the cell death induced by the UV-excited NPs of TiO_2_ or ZnO is played by ROS. They are strong oxidants but also important agents modulating ROS-sensitive signaling pathways that lead to the cell death. Biola-Clier et al. [145] report the ROS-mediated downregulation of DNA repair processes such as base excision repair and nucleotide excision repair. Toyooka et al. [146], when examining toxicity of TiO_2_ NPs (15 μg mL^−1^) to human A549 cells, concluded there was an increased H2AX histone phosphorylation that followed the oxidative stress-induced DNA double-strand breaks. Similar results obtained Premanathan et al. [147], having examined human HL-60 cells exposed to ZnO NPs (10 μg mL^−1^). Phosphorylated H2AX (γH2AX) histone is considered to be a marker of DNA damages [148]. Its phosphorylation is caused by ataxia telangiectasia mutated kinase (ATM; EC 2.7.11.1), as well as by ataxia telangiectasia and Rad 3-related kinase (ATR; EC 2.7.11.1), enzymes participating in the DNA damage response (DDR) pathway. The key effector protein on this pathway is p53. The importance of the ROS-induced activation of p53-mediated DNA damage check point signals in apoptosis of TiO_2_ NP-treated human peripheral blood lymphocytes (PBL) was stressed by Kang et al. [143]. Responding to the big scale of the DNA damages, p53 induces the pro-apoptotic protein gene expression, followed by the cell death [143] (Fig. 3).

Yoo et al. [144] studied the ROS mediation in the induction of both intrinsic and extrinsic apoptotic pathways since ROS stimulate the increase of pro-apoptotic protein (Bax) and death ligand (FasL) levels, respectively. ROS induce apoptosis in tumor cells also by modification of the activity of mitogen-activated protein kinases (MAPKs; EC 2.7.11.24) [149] and of the nuclear factor kappa B (NF-κB) [150]. Cheng et al. [151] report that PDT-generated ROS modify the ability of thioredoxin (Trx), the intracellular sensor of oxidative stress, to inhibit the apoptosis signal-regulating kinase 1 (ASK1; EC 2.7.11.25). ASK1 forms an inactive structure with reduced Trx. In the presence of ROS, the oxidation of Trx takes place and the structure degrades. The free form of ASK1 phosphorylates MAPKs such as c-Jun N-terminal protein kinase isoform 1 and 2 (JNK1/2), p38 mitogen-activated protein kinases (p38 MAPKs), and extracellular signal-regulated kinase isoform 1 and 2 (ERK1/2) [152]. Phosphorylated MAPKs translocate into the nucleus, where they induce the formation of the activator protein 1 (AP-1), a transcription factor regulating the expression of the AP-1-dependant pro- and anti-apoptotic genes [153]. JNK1/2 and p38 MAPKs lead to AP-1-mediated inactivation of anti-apoptotic proteins (Bcl-2 and Bcl-xL), the result of which is the formation of apoptosome and the activation of caspases cascade, both subsequently leading to the proteolysis of inhibitor of caspase-activated DNase (ICAD) and then, as a consequence, to the DNA fragmentation [154, 155]. JNK1/2 and p38 MAPKs promote apoptosis in case the ERK1/2 are inhibited or remain inactive [156, 157]. This conclusion was confirmed by the studies on the cytotoxicity of ZnO NPs to human HepG2 cells performed by Yuan et al. [158]. Nevertheless, in oxidative stress conditions, ERK1/2 might adopt a pro-apoptotic role since they attenuate the activity of protein kinase B (PKB, EC 2.7.11.1), called also AKT1 [159]. This phenomenon was observed in renal proximal tubule epithelial cells (LLC-PK1 line) [160]. AKT1 activates NF-κB through IκB kinase (IKK; EC 2.7.11.10) phosphorylation [161]. Coupienne et al. [162] suggest that ROS induce the AKT-mediated inactivation of NF-κB, an inhibitor of apoptosis. The NF-κB activation occurs in the cytoplasm and follows the detachment of the κB inhibitor (IκB). The active form of NF-κB translocates into the nucleus, where it initiates expression of genes that encode anti-apoptotic proteins (Fig. 3). The studies by Granville et al. [163] revealed PDT-treated human promyelocytic leukemia cells (HL-60 line) showed no detectable IκB level in the cytoplasm. It might constitute an explanation of typical morphological symptoms of the apoptotic cell death, as noticed using the confocal laser scanning microscope (CLSM), such as nucleus fragmentation, cytoplasm shrinkage, apoptotic bodies formation, or lack of inflammation reaction.

PDT-induced damages of the endoplasmic reticulum habitually result in the cell death by autophagy [164] (Fig. 3). In the oxidative stress, the activation of JNK1 that phosphorylates Bcl-2 occurs. Consequently, the dissociation of Beclin 1/Bcl-2 complex, hence the activation of Beclin 1, a pro-autophagic protein, takes place [165–167]. Such effects were noticed in PDT-treated human prostate adenocarcinoma cells (DU-145 and PC3 lines) [168]. Beclin 1 inhibits apoptosis by the inactivation of caspase-8 (CASP-8; EC 3.4.22.61), an enzyme transforming Bid to tBid, a pro-apoptotic protein. Beclin 1 also contributes, along with the phosphatidylinositol 3-kinase class III (PI3K3C; EC 2.7.1.137), towards the formation of the autophagosome that eliminates cytoplasmic “waste” material [166]. Autophagosome fuses with lisosome, leading therefore to the formation of autophagolisosome, in which lysosomal hydrolases complete the process of the definite destruction of its content [169–171]. Among the morphological symptoms of the autophagic cell death, there are increased number of autophagic vesicles, the degradation of Golgi apparatus, and endoplasmic reticulum, as well as no inflammation reaction [164]. Those symptoms were observed by Yu et el. [128] in TiO_2_ NP-treated human bronchial epithelial cells (16HBE14o-line). High level of ROS in a cell results in the increase of the AMP/ATP ratio, an effect stimulating the AMP-activated protein kinase (AMPK; EC, 2.7.11.31) [172, 173]. AMPK may efficiently inhibit the mammalian target of rapamycin kinase (mTOR; EC. 2.7.11.1). mTOR is a key autophagy inhibitor and is frequently overexpressed in miscellaneous tumor cells [174, 175]. Its PDT-induced inhibition, leading up to the autophagy, was observed in human breast adenocarcinoma cells (MDA-MB-231 line) [176].

High level of the PDT-generated ROS, inflicting pro-apoptotic (Bax, Bak) and pro-autophagic (Beclin 1) protein damages, as well as plasma membrane disruption, results in a cell death by necrosis [177, 178] (Fig. 3). Cell membrane oxidative damages cause the increase of the cytosolic Ca^2+^ level (>1 μM) that activates Ca^2+^-dependent proteases. Other effects are dissipation of mitochondrial membrane potential (Δψ_m_) and a dramatic decrease of the intracellular ATP level [178]. Guo et al. [179, 180] exposed rat retinal ganglion cells (RGC-5 line) to ZnO NPs and concluded there was a remarkable increase in Ca^2+^ level in cytosol that resulted from the downregulation of plasma membrane calcium ATPase isoform 1 (PMCA1; EC 3.6.3.8) activity. Disrupted homeostasis of Ca^2+^ was also observed in human lens epithelial cells (HLE-B3 line) incubated with ZnO NPs and, subsequently, exposed to UV-B radiation [181]. Typical morphological symptoms of the necrotic cell death encompass: cell swelling, destruction of nucleus and of other cell organelles, plasma membrane disruption–all of them resulting in a cell disintegration, followed by the release of the cell content into the intercellular space hence inflammation reaction [182]. Xu et al. [108] described the necrosis of human lung adenocarcinoma cells (APC-A1 line), induced by photoexcited TiO_2_ NPs.

PDT-treatment of tumor cells, using the metal oxide nanoparticles, results in the cell death, presenting the consequence of irreversible DNA damage [146], plasma membrane disintegration [183], intracellular Ca^2+^ homeostasis disruption [179, 180], strong reduction or complete exhaustion of the redox compound pool, essential in ATP synthesis [184]. The main factor of reported damages is PDT-induced oxidative stress [184–187].

Nanocomposites, e.g., gold-doped TiO_2_ (Au/TiO_2_) [101], silver-doped TiO_2_ (Ag/TiO_2_) [102], platinum-doped TiO_2_ (Pt/TiO_2_) [188], or silver-doped ZnO (Ag/ZnO) [189] are compounds increasingly often applied in anticancer therapy. The conjugation of NPs of a precious metal with TiO_2_ NPs increases the catalytic activity of this oxide, a phenomenon confirmed in the studies of Abdulla-al-Mamun et al. [102]. They reported that Ag/TiO_2_ nanocomposite showed an 80% higher effectiveness in eradication of human HeLa cells than TiO_2_ NPs. In other studies, TiO_2_ NPs were conjugated with folic acid (FA) molecules [190]. Such conjugation enhanced the ability of human HeLa cells to uptake FA/TiO_2_ nanocomposite by FA receptors in plasma membrane and, therefore, to leverage the efficiency of cancer cells eradication. Feng et al. [191] confirmed high biocompatibility of FA/TiO_2_ nanocomposite and its incorporation into human nasopharyngeal carcinoma (KB) cells. Rozhkova et al. [109] designed TiO_2_-based nanocomposites that target and eradicate brain carcinoma cells in vivo with no harm to adjacent healthy cells. IL13α2R antibodies were conjugated with TiO_2_ NPs through 3,4-dihydroxyphenylacetic acid (DOPAC). The antibodies recognize cancer cells, bind to them and are eventually uptaken by means of phagocytosis. In the process of tumor cells exposition to UV-A radiation, TiO_2_-based nanoconstructs generate the formation of ROS that subsequently damage the cancer cells and induce their apoptosis. The selection of suitable antibodies can help design nanocomposites able to precisely target and eradicate many types of cancer cells [82, 83]. Xu et al. [100] advocate a simultaneous application of conjugates of TiO_2_ NPs with monoclonal antibodies, along with reversible plasma membrane injury by means of electric field, so called electroporation. As the authors suggest, such combination may increase the accuracy of cancer cell recognition by TiO_2_-based nanocomposites and, in consequence, enhance the TiO_2_-based nanoconstructs penetration into cytosol. Using that method, it was possible to eradicate in vitro all human LoVo cells that were subject to experiment, within merely 90 min.

One of the main disadvantages of PDT is the limited penetration depth of the tissues by the UV radiation. Therefore, this method is suitable to eradicate tumor cells located in superficial depths [192]. This shortcoming might be rectified, and deeper located tumor cells can still be eradicated using PDT, if NPs of TiO_2_ and ZnO are conjugated with upconverting nanoparticles (UCNPs) [193]. Ytterbium and erbium doped sodium yttrium fluoride (NaYF4:Yb,Er) is frequently applied as transducer [194]. UCNPs absorb near infrared (NIR, 700 ≤ λ < 850 nm) radiation and emit UV (λ < 400 nm) radiation. NIR radiation, compared to UV radiation, penetrates tissues in bigger depths but is also less harmful to the organism. Therefore, UCNP-based PDT gains enhanced effectiveness in the eradication of deeper depths tumor cells [195]. This finding has been confirmed both in vitro, in human cancer cells (HeLa and MCF-7 lines) and in vivo, in female Balb/c nude mice [196, 197].

Thevenot et al. [198] withdrew from TiO_2_ NPs excitation by UV radiation in favor of the chemical functionalization of their surfaces by binding to them –OH, –NH_2_, or –COOH groups. Then, they examined TiO_2_ NPs, as modified on their surface, in respect of their influence on the viability of a number of cancer cell lines (e.g., B16F10 mouse melanoma cell line, 3T3 mouse embryonic fibroblast cell line, or PC-3M human prostate carcinoma cell line) in vitro. According to the authors, the viability of the examined cancer cell lines was dependent upon both the concentration of TiO_2_ NPs in the examined cells and the type of functional group altering the surface properties of TiO_2_ NPs. The highest cyto- and genotoxicity exhibited NPs functionalized by –OH groups. The design of surface properties of NMs and NPs presents a valuable tool in the targeted cancer therapy.

#### Sonodynamic Therapy

Limited possibilities to apply PDT in the cancer therapy gave rise to seek different cancer treatment modalities. An example of an alternative tumor eradication method is SDT. This therapy is based on a synergistic effect on a cancer cell by sonosensitizer and US [199]. US can activate particles of compounds called sonosensitizers. In comparison with UV or NIR radiation, US penetrates tissues much more effectively as it reaches deeper depths tumor cells. The introduction of sonosensitizers into cancer cells and their subsequent exposure to US in order to generate ROS is the underlying factor of SDT [199, 200] (Fig. 2).

Harada et al. [114] showed in their studies in vitro that there was a remarkable reduction (in excess of 50%) in viability of human melanoma cells (C32 line) upon exposure to the TiO_2_ NP-based SDT (1 MHz, 30 s). Moosavi Nejda et al. [200] examined in vitro the influence of the US-excited TiO_2_ NPs (100 μg mL^−1^) on the viability of human oral squamous carcinoma cells (HSC-2 line). The authors proved the increased intensity of US (20, 32, 55, 73 W cm^−2^) and the prolonged sonication period (0.1, 1.0, 3.0 s) enhanced the damage ratio in the human HSC-2 cells, finally leading to their necrosis at the conditions of 73 W cm^−2^ and 3 s. Yamaguchi et al. [183] believe that TiO_2_ NP-based cancer therapies will soon become a valuable tool in fighting malignant gliomas. The authors compared PDT to SDT in respect of their effectiveness in eradication of human glioblastoma cells (U251 line). With PDT, the toxicity effect of TiO_2_ NPs (20 μg mL^−1^) was noticeable after 2-h exposure of single cancer cell layer to UV-A radiation (5.0 mW cm^−2^). Moreover, the effect was fairly restricted by free radical scavengers. Cytotoxicity of TiO_2_ NPs (20 μg mL^−1^) in case of SDT was proportional to the sonication period, and it was not dependent on antioxidants concentration, an observation indicating there must be a different mechanism of intracellular structure damages. US, propagating in cell, induce the formation of cavitation bubbles which free energy as they implode. It is assumed that acoustic cavitation can not only lead to sonosensitizer excitation, the result of which is ^1^O_2_ formation, but it can also cause—with no ROS involved—a disruption in plasma membrane integrity. Saito et al. [201] report that the necrotic death of human corneal endothelial cells after the US exposure can take place rapidly and could be attributed to mechanically induced damage to the plasma membrane. Thanks to fluorescent dyes, a greater amount of plasma membrane damages resulting from the TiO_2_ NP-based SDT could be noticed than in the TiO_2_ NP-based PDT.

In SDT, similarly to PDT, TiO_2_ NPs are also conjugated with precious metals or antibodies. The subject of studies conducted by Ogino et al. [202], as well as Ninomiya et al. [203, 204], was the examination of TiO_2_ NP toxicity to human HepG2 cells. To increase the TiO_2_ NPs uptake rate by cancer cells, pre-S1/S2, a model antibody recognizing hepatocytes, was conjugated with the TiO_2_ NPs. The ability of such conjugates to recognize cancer cells was examined using the surface plasmon resonance and immunofluorescence techniques. Apoptosis of some cells of the human HepG2 cells in vitro was firstly observed after 6 h from the termination of the TiO_2_ NP sonication (1 MHz, 30 s), and after the following 90 h, the number of dividing cells decreased by 45%. The studies conducted by Ninomiya et al. [205] revealed the high efficiency of the TiO_2_ NP-based SDT as a tool to reduce the proliferation of human MCF-7 cells, too. In this case, avidin was immobilized on the surface of TiO_2_ NPs in order to enhance its uptake rate by cancer cells. Within 96 h after the termination of the TiO_2_ NP sonication (1 MHz, 30 s), the number of viable cancer cells was reduced by 30%.

TiO_2_ and ZnO NPs are excellent sensitizers and oxidizing agents. The feature of the ROS generated on their surface is a higher oxidation potential as well as a short half-life (<0.04 μs) [41]. Therefore, the occurrence of ROS-induced oxidative injuries is limited to a small distance (<0.02 μm) from the subcellular position of the sensitizer NPs [54]. NPs administered into the bloodstream become covered by a plasma proteins layer, called protein corona, that lowers their cytotoxicity and, depending on the type of adsorbed plasma proteins, modulates their immunotoxicity and the targeted delivery into tumor cells [206, 207]. Garvas et al. [208] proved the cytotoxicity of TiO_2_ NPs in vivo is limited by serum proteins covering their surface that exhibit a trapping ability of photogenerated ROS. The knowledge on the potential application of NPs of TiO_2_ and ZnO in therapy and diagnostics of neoplastic maladies is continuously growing. Nonetheless, further studies are necessary to improve the tumor targetability of TiO_2_ and ZnO NPs, as well as to increase the therapeutic window of PDT using metal oxide NPs [209, 210].

## Conclusions

Traditional methods of cancer therapy inflict a series of side effects, including the impairment of the immune system. Recent years have witnessed many efforts in creating alternative cancer therapy methods that would be less invasive and would exhibit a site-specific activity. PDT might present a viable solution. Among photosensitizers that have been thoroughly examined in view of their potential application in cancer therapies, there are TiO_2_ and ZnO in their NPs form. UV-excited NPs of TiO_2_ and ZnO are a source of ROS that form on their surfaces. ROS are agents with a high oxidative potential that damage primarily DNA and cell membranes of cancer cells. Plasma membrane damages lead to cell necrosis, nucleus and mitochondria damages induce apoptotic cell death, and endoplasmic reticulum damages cause cell death by autophagy. Limited tissue penetration ability of UV radiation presents a serious obstacle in the application of UV-sensitizing NPs of TiO_2_ and ZnO in cancer therapy. Efforts to apply NIR radiation, since it is less harmful and can penetrate deeper depths tissues, have so far not been successful. Therefore, a new method had to be set up, allowing to generate ROS in deeper depths tissues. Such a method is SDT, using US with low tissue attenuation coefficient. However, SDT exhibits one major disadvantage: insufficient tumor targetability by NPs of TiO_2_ and ZnO. The difficulty in preparation of a drug formula that would allow the targeted delivery of NPs to cancer cells is an important factor that restricts the application of photo- and sonosensitizers in cancer therapy. Thanks to the development of nanotechnology, various drug delivery systems in organisms have been designed. One of them is compiling conjugates of TiO_2_ or ZnO in their NPs form with monoclonal antibodies, a method that allows targeting the photo- and sonosensitizers on a selected receptor. The design of modern, more effective cancer fight methods remains an important challenge to contemporary medicine. The scientists are still facing many years of studies on how to optimize cancer therapy using nanotechnology whereas the results have so far been fairly promising.

## Abbreviations

AMPK: AMP-activated protein kinase
AP-1: Activator protein 1
Apaf-1: Apoptotic protease-activating factor 1
ASK: Apoptosis signal-regulating kinase
ATM: Ataxia telangiectasia mutated kinase
ATR: Ataxia telangiectasia Rad 3-related kinase
BAL: Bronchoalveolar lavage
CASP: Caspase
CAT: Catalase
CB: Conduction band
CLSM: Confocal laser scanning microscope
DDR: DNA damage response
DOPAC: 3,4-Dihydroxyphenylacetic acid
ERK: Extracellular signal-regulated kinase
FA: Folic acid
GPX: Glutathione peroxidase
GSH: Glutathione
ICAD: Inhibitor of caspase-activated DNase
IKK: κB inhibitor kinase
IκB: κB inhibitor
JNK: c-Jun N-terminal protein kinase
LPOs: Lipid hydroperoxides
MAPKs: Mitogen-activated protein kinases
MPs: Microparticles
mTOR: Mammalian target of rapamycin kinase
NF-κB: Nuclear factor-kappa B
NMs: Nanomaterials
NPs: Nanoparticles
NRRT: Neutral red retention time
PDT: Photodynamic therapy
PI3K3C: Phosphatidylinositol 3-kinase class III
PKB: Protein kinase B
PMCA: Plasma membrane calcium ATPase
ROS: Reactive oxygen species
SCGE: Single-cell gel electrophoresis
SDT: Sonodynamic therapy
SOD: Superoxide dismutase
Trx: Thioredoxin
US: Ultrasound
UV: Ultraviolet
VB: Valence band
